# Isolation and identification of *Acanthamoeba* strains from soil and tap water in Yanji, China

**DOI:** 10.1186/s12199-017-0655-2

**Published:** 2017-06-30

**Authors:** Yinghua Xuan, Yanqin Shen, Yuxi Ge, Gen Yan, Shanzi Zheng

**Affiliations:** 1 Department of Basic Medicine, Medical School, Jiangnan University, 1800 Li Hu Road, Wuxi, 214122 Jiangsu China; 2 Department of Radiology, Affiliated Hospital of Jiangnan University, No. 200 Huihe Road, Wuxi, 214062 Jiangsu China; 3 Department of Parasitology, College of Medicine, Yanbian University, Yanji, 133000 Jilin China

**Keywords:** *Acanthamoeba*, Environment, Genotype, 18S rDNA, Mitochondrial DNA RFLP

## Abstract

**Background:**

Members of the genus *Acanthamoeba* are widely distributed throughout the world, and some of them are considered pathogenic, as they are capable of causing corneal and central nervous system diseases. In this study, we isolated *Acanthamoeba* strains from soil and tap water in Yanji, China.

**Methods:**

We identified four strains of *Acanthamoeba* (CJY/S1, CJY/S2, CJY/S3, and CJY/W1) using mitochondrial DNA restriction fragment length polymorphism (mtDNA RFLP) analysis. Nuclear 18S rDNA sequences were used for phylogenetic analysis and species identification.

**Results:**

Genotypic characterization of the isolates showed that they belonged to genotypes T4 (CJY/S1 and CJY/S2), T5 (CJY/S3), and T16 (CJY/W1). Sequence differences between CJY/S1 and *Acanthamoeba castellanii* Neff, CJY/S2 and *Acanthamoeba* KA/E7, and CJY/S3 and *Acanthamoeba lenticulata* 68–2 were 0.31, 0.2, and 0.26%, respectively. 18S ribosomal deoxyribonucleic acid (rDNA) of CJY/W1 had 99% sequence identity to that of *Acanthamoeba* sp. U/H-C1. Strains CJY/S1 and CJY/S2, isolated from soil, had similar mtDNA RFLP patterns, whereas strain CJY/W1, isolated from tap water, displayed a different pattern.

**Conclusions:**

To the best of our knowledge, this is the first report on the identification of genotypes T4, T5, and T16 from environmental sources in Yanji, China.

## Background

*Acanthamoeba* species are widely distributed in the environment: their habitats include soil, freshwater, seawater, dust, and putrilage. Some *Acanthamoeba* species can cause keratitis, granulomatous amoebic encephalitis (GAE), pulmonary infections, cutaneous lesions, rhinosinusitis, osteomyelitis, or diffuse inflammation [1–3]. *Acanthamoeba* keratitis can lead to scarring of the cornea, resulting in a permanent visual impairment or complete blindness. GAE is a central nervous system disease that usually occurs in immunocompromised patients, such as those with acquired immune deficiency syndrome, systemic lupus erythematosus, and transplanted organ, or those undergoing chemotherapy for cancer. Nonetheless, several cases of GAE and skin infection due to *Acanthamoeba* spp. have been reported in immunocompetent individuals [4–8].

Eye infections have been reported to be caused by *Acanthamoeba castellanii*, *Acanthamoeba polyphaga*, *Acanthamoeba rhysodes*, *Acanthamoeba culbertsoni*, *Acanthamoeba lugdunensis*, *Acanthamoeba griffini*, *Acanthamoeba hatchetti*, *Acanthamoeba quina*, *Acanthamoeba lenticulata*, and *Acanthamoeba triangularis*. Central nervous system diseases have been caused by *A. castellanii*, *A. culbertsoni*, *Acanthamoeba astronyxis*, *A. rhysodes*, *Acanthamoeba healyi*, and *A. lenticulata* [6, 9–13].

There have been few reports of *Acanthamoeba* spp. isolated from environmental samples in China [14]. In this study, we report the molecular biological characterization of four strains of *Acanthamoeba* (CJY/S1, CJY/S2, CJY/S3, and CJY/W1) isolated from soil and tap water in China. We identified these species by using mitochondrial DNA restriction fragment length polymorphism (mtDNA RFLP) analysis and 18S rDNA sequence alignment. We found that the three strains isolated from the soil belonged to the morphological group II and had genotypes T4 and T5, whereas the strain isolated from tap water belonged to the morphological group II and had genotype T16.

## Methods

### Isolation and cultivation of *Acanthamoeba*

Samples of soil and tap water were collected in Yanji, China. The samples were loaded onto 1.5% agar plates covered with heat-inactivated (60 °C for 1 h) *Escherichia coli* (American Type Culture Collection, ATCC 25922, free of plasmid). The plates were incubated at 25 °C, and growth of *Acanthamoeba* was observed under an inverted microscope on a daily basis for 1 week. Each cyst isolated with a glass capillary was inoculated on a new agar plate and incubated for 1 week. For axenization, a piece of agar (1 cm × 1 cm) covered with cysts was treated with 0.1 N HCl for 24 h, washed three times with sterile water, placed in peptone yeast glucose medium (10 g proteose peptone, 10 g yeast extract, 10 mL of 50% glucose, 10 mL of 0.5 M Na_2_HPO_4_, and 10 mL of 0.5 M K_2_HPO_4_ in 970 mL of sterile water), and incubated at 25 °C for 3 weeks. When most of the amoebae reached trophozoite stage, they were harvested and washed three times with phosphate-buffered saline.

### Morphological examination

A cyst formed on the monoxenic plate was picked with a sterilized inoculating loop and transferred to a glass slide with a drop of sterile distilled water. The slide was then covered with a coverslip. Fifty cysts per plate were observed and measured under a Nomarski (differential interference contrast) microscope (Olympus, Japan).

### Analysis of 18S rDNA sequences

Genomic DNA was extracted using phenol/chloroform method. The 18S rRNA gene was amplified using the following primers by Xuan et al. [13]: forward, 5′-CCGAATTCGTCGACAACCTGGTTGATCCTGCCAGT-3′; reverse, 5′-GGATCCAAGCTTGATCCTTCTGCAGGTTCACCTAC-3′. The amplified products were resolved by electrophoresis, recovered from the gel, and ligated into a T/A cloning vector (pGEM-T Easy Vector System I, Promega, USA) for subsequent transformation of *E. coli*. Positive clones were picked, and recombinant plasmid DNA was extracted using the Wizard® Plus Minipreps DNA Purification System (Promega, USA). Plasmids with inserts of the correct size were identified by *Eco*RI digestion and sequenced. The obtained final 18S ribosomal DNA (rDNA) sequences of the *Acanthamoeba* strains were deposited in GenBank (accession nos. KY827389–KY827392). The sequences were then compared with those of other *Acanthamoeba* sequences in GenBank using the Basic Local Alignment Search Tool (BLAST) search engine. Clustal X and GeneDoc were used for pairwise alignment and calculation of percent sequence dissimilarity. Phylogenetic analyses were performed, and the phylogenetic tree was drawn using the neighbor-joining (NJ) method with MEGA3 [15]. The reference strains of *Acanthamoeba* and the GenBank accession numbers of the 18S rDNA sequences used in this study are as follows: *A. castellanii* CDC:0981:V006 (T1), U07400; *Acanthamoeba palestinensis* (T2), U07411; *A. griffini* H37 (T3), S81337; *A. castellanii* Neff (T4), U07416; *A. lenticulata* E18-2 (T5), U94735; *A. palestinensis* 2802 (T6), AF019063; *A. astronyxis* R&H (T7), AF019064; *Acanthamoeba tubiashi* OC-15C (T8), AF019065; *Acanthamoeba comandoni* Pussard (T9), AF019066; *A. culbertsoni* A-1 (T10), AF019067; *A. hatchetti* BH-2 (T11), AF019068; *A. healyi* OC-3A (T12), AF019070; *Acanthamoeba* sp. UWC9 (T13), AF132134; *Acanthamoeba* sp. PN13 (T14), AF333609; *Acanthamoeba jacobsi* ATCC 30732 (T15), AY262360; *Acanthamoeba* sp. U/H-C1 (T16), AY026245; *Acanthamoeba* sp. E1a (T17), GU808277; *Acanthamoeba byersi* CDC:V621 (T18), KC822464; *Acanthamoeba micheli* BRO-2 (T19), KP711387; and *Acanthamoeba* sp. OSU 04-020 (T20), DQ451161.

### Extraction of mtDNA and RFLP analysis

mtDNA of *Acanthamoeba* isolates was extracted using the method described by Yagita and Endo [16]. Briefly, amoebae were harvested and washed in cold phosphate-buffered saline, treated with TEG buffer (25 mM Tris–HCl, 10 mM ethylenediaminetetraacetic acid (EDTA), 50 mM glucose, pH 8.0), lysed with a fresh solution of 1% sodium dodecyl sulfate in 0.2 N NaOH and potassium acetate buffer, and left on ice for 30 min. mtDNA was then extracted with a phenol/chloroform mixture (1:1) and recovered by the precipitation with cold absolute ethanol in the presence of sodium acetate. The extracted mtDNA was then digested with *Eco*RI at 37 °C. The digested mtDNA was electrophoresed in 0.7% agarose gel and stained with ethidium bromide. The mtDNA RFLP patterns were observed and photographed.

## Results

### Morphology of *Acanthamoeba* isolates

**Fig. 1:**
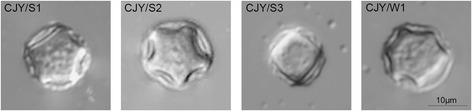
Photomicrographs of cysts of the *Acanthamoeba* isolates CJY/S1, CJY/S2, CJY/S3, and CJY/W1. Images were taken under a bright-field inverted microscope. *Bar* = 10 μm

Cysts of the *Acanthamoeba* isolates CJY/S1, CJY/S2, CJY/S3, and CJY/W1 exhibited morphological characteristics typical of group II, as defined by Pussard and Pons [17]. They exhibited double-walled cyst morphology and featured thick, wrinkled ectocysts and satellite or polygonal endocysts (Fig. 1). The cyst diameter varied from 12.0 to 18.8 μm, and the number of arms was four to six (Table 1).

**Table 1:** Morphological feature of *Acanthamoeba* isolates analyzed in this study

Isolates	Cyst diameter* (average)	No. of arms (average)
CJY/S1 CJY/S2	14.5 (12.3~17.2) 15.1 (13.1~17.8)	4.1 (4~6) 4.5 (4~6)
CJY/S3	13.3 (12.0~15.3)	4.3 (4~5)
CJY/W1	15.3 (12.5~18.8)	4.6 (4~6)

* Unit: µm

### 18S rRNA sequence analysis of the four isolated *Acanthamoeba* strains

**Fig. 2:**
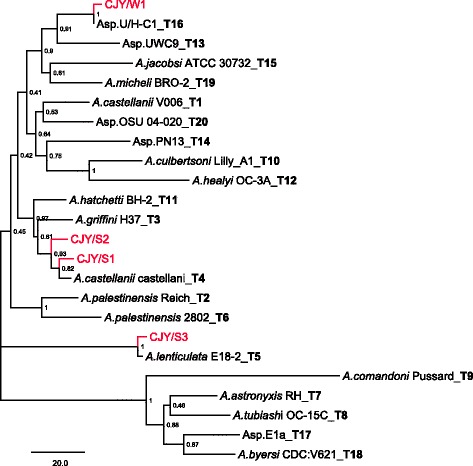
Dendrogram of *Acanthamoeba* strains based on 18S rDNA sequences. The neighbor-joining tree reflects the affiliation of the *Acanthamoeba* sp. strains CJY/S1, CJY/S2, CJY/S3, and CJY/W1 with the reference strains of genotypes T1–T20. T1: *A. castellanii* CDC: 0981:V006, GenBank accession no. U07400; T2: *A. palestinensis*, U07411; T3: *A. griffini* H37, S81337; T4: *A. castellanii* Neff, U07416; T5: *A. lenticulata* E18-2, U94735; T6: *A. palestinensis* 2802, AF019063; T7: *A. astronyxis*, AF019064; T8: *A. tubiashi* OC-15C, AF019065; T9: *A. comandoni*, AF019066; T10: *A. culbertsoni* A-1, AF019067; T11: *A. hatchetti* BH-2, AF019068; T12: *A. healyi*, AF019070; T13: *Acanthamoeba* sp. UWC9, AF132134; T14: *Acanthamoeba* sp. PN13, AF333609; T15: *A. jacobsi* ATCC 30732, AY262360; T16: *Acanthamoeba* sp. U/H-C1, AY026245; T17: *Acanthamoeba* sp. E1a, GU808277; T18: *A. byersi* CDC:V621, KC822464; T19: *A micheli* BRO-2, KP711387; T20: *Acanthamoeba* sp. OSU 04–020, DQ451161

The 18S rRNA genes of the *Acanthamoeba* sp. strains CJY/S1, CJY/S2, CJY/S3, and CJY/W1 were amplified using primers specific for *Acanthamoeba* spp. The polymerase chain reaction (PCR) products were cloned into the pGEM-T vector, and recombinant plasmids were digested with *Eco*RI. The full lengths of the 18S rRNA genes of the *Acanthamoeba* sp. strains CJY/S1, CJY/S2, CJY/S3, and CJY/W1 were 2255, 2252, 2292, and 2252 bp, respectively. *Acanthamoeba* sp. CJY/S1 and CJY/S2 had very high 18S rDNA sequence similarity with 18S rDNA sequences of *A. castellanii* Neff (99.82%) and *Acanthamoeba* sp. KA/E7 (99.69%). *Acanthamoeba* sp. CJY/S3 showed very high 18S rDNA sequence similarity with that of *A. lenticulata* 68–2 (99.74%). The 18S rRNA gene sequence of *Acanthamoeba* sp. CJY/W1 was closely related to the sequences of *Acanthamoeba* sp. U/H-C1 (99%) and *Acanthamoeba* sp. UWC9 (95%). Sequence alignment showed that 18S rDNA sequences of *Acanthamoeba* sp. strains CJY/S1 and CJY/S2 corresponded to genotype T4 and CJY/S3 corresponded to genotype T5, whereas the sequence of *Acanthamoeba* sp. CJY/W1 corresponded to genotype T16 (Fig. 2).

### mtDNA RFLP

**Fig. 3:**
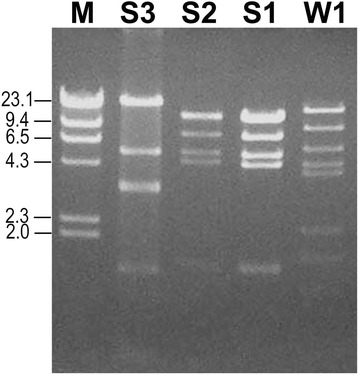
Restriction fragment patterns of *Eco*RI-digested mitochondrial DNA of *Acanthamoeba* sp. strains CJY/S1, CJY/S2, CJY/S3, and CJY/W1. The DNA fragments were separated on 0.7% agarose gel

Figure 3 shows agarose gel electrophoretic patterns of *Eco*RI-digested mtDNA extracted from the *Acanthamoeba* sp. strains CJY/S1, CJY/S2, CJY/S3, and CJY/W1. *Acanthamoeba* sp. strains CJY/S1 and CJY/S2 showed extremely similar mtDNA RFLP patterns, whereas *Acanthamoeba* sp. CJY/S3 and CJY/W1 each displayed a different pattern.

## Discussion

There are many species of free-living amoebae. Some, such as *Acanthamoeba* spp., *Naegleria* spp., *Hartmannella* spp., or *Balamuthia mandrillaris*, are opportunists that can cause infections in humans and animals [18, 19]. *Naegleria* and *Acanthamoeba* species have been identified as causes of serious human infections. Some species of *Acanthamoeba* can cause amoebic keratitis, particularly in contact lens wearers and immunocompromised individuals experiencing subacute or chronic central nervous system infections [20, 21].

Eighteen species of *Acanthamoeba* have been classified into three groups according to the shape and size of cysts. Species in group I are nonpathogenic except *A. astronyxis*, *A. byersi* and *A. comandoni* [22, 23]. Most of the pathogenic *Acanthamoeba* species belong to group II. Species in group III, such as *A. culbertsoni*, *A. healyi*, and *A. lenticulata*, often cause infections of the brain. The cyst morphology of the four isolates characterized in this study resembled that of various species within morphological group II. However, the classification of *Acanthamoeba* spp. based on morphological characteristics has proven to be unreliable. The morphology of *Acanthamoeba* spp. may change depending on culture conditions. Furthermore, different *Acanthamoeba* species in the same group can have similar morphology, and *Acanthamoeba* cysts of two species may show only transient differences, thereby causing difficulties in the identification of the species.

Lass et al. reported partial sequences of T4 strains in environmental samples in China recently [14], which is different from our data which is full of 18S sequences of four distinct genotypes of isolated strains. At present, sequence analysis of genomic DNA is considered the method of choice for identifying species of *Acanthamoeba*. Sequence analysis of 18S rRNA genes is frequently used.

Based on the nucleotide sequence of 18S rDNA, *Acanthamoeba* was initially clustered into 12 genotypes, from T1 to T12 [12, 24]. Recently, new genotypes of *Acanthamoeba* have been identified [25–29]. Five genotypes of *Acanthamoeba* are associated with keratitis: T4 is the primary genotype and T3 is the secondary genotype, whereas *Acanthamoeba* species of the remaining genotypes (T5, T6, and T2) are considered to be rare causes of this disease. Gast [28] reported that sequence differences among 15 strains of *Acanthamoeba* within genotype T4 were in the range of 0–4%, whereas sequence differences among genotypes were 6–12%.

In this study, the full-length 18S rRNA genes from the *Acanthamoeba* sp. strains CJY/S1, CJY/S2, CJY/S3, and CJY/W1, isolated from soil and tap water of Yanji, China, were determined to be 2255, 2252, 2292, and 2252 bp, respectively. These lengths are close to the range of 2300–2700 bp reported by Stothard et al. [12]. The 18S rDNA sequences of the *Acanthamoeba* sp. strains CJY/S1, CJY/S2, CJY/S3, and CJY/W1 were compared with those of reference strains of genotypes T1–T20, which were obtained from GenBank by using BLAST searches. Pairwise alignment and calculation of the percent sequence dissimilarity using Clustal X and GeneDoc showed that *Acanthamoeba* sp. strains CJY/S1 and CJY/S2 had genotype T4, which encompasses the majority of clinical and environmental isolates of *Acanthamoeba*. In a phylogenetic tree based on the 18S rDNA sequence, *Acanthamoeba* CJY/S3 strain was positioned close to genotype T5 species and was related to *A. lenticulata*. The *Acanthamoeba* sp. strains CJY/S1 and CJY/S2 showed extremely similar mtDNA RFLP patterns. *Acanthamoeba* sp. CJY/W1 had genotype T16 and was closely related to *Acanthamoeba* sp. U/H-C1 (99%) and *Acanthamoeba* sp. UWC9 (95%) [30]. *Acanthamoeba* sp. CJY/W1, isolated from tap water, had a mtDNA RFLP pattern different from those of the *Acanthamoeba* sp. strains CJY/S1, CJY/S2, and CJY/S3, which were isolated from soil.

Recent studies have shown that *A. castellanii* (genotype T4) and *A. lenticulata* (genotype T5) can infect the cornea and central nervous system in humans, but there are insufficient reports pertaining to strains of genotype T16. Further research is required to determine whether the *Acanthamoeba* sp. strains CJY/S1, CJY/S2, CJY/S3, and CJY/W1 isolated by us from environmental samples could be pathogenic to humans and animals.

## Conclusions

Strains CJY/S1 and CJY/S2, isolated from soil, had similar mtDNA RFLP patterns, whereas strain CJY/W1, isolated from tap water, displayed a different pattern. To the best of our knowledge, this is the first report on the identification of genotypes T4, T5, and T16 from environmental sources in Yanji, China.

## Abbreviations

ATCC: American Type Culture Collection
BLAST: Basic Local Alignment Search Tool
EDTA: Ethylene Diamine Tetraacetic Acid
GAE: Granulomatous amoebic encephalitis
mtDNA RFLP: Mitochondrial DNA restriction fragment length polymorphism
PCR: Polymerase chain reaction
rDNA: ribosomal deoxyribonucleic acid
